# REVISE: re-evaluating the inhibition of stress erosions in the ICU—statistical analysis plan for a randomized trial

**DOI:** 10.1186/s13063-023-07794-z

**Published:** 2023-12-06

**Authors:** Diane Heels-Ansdell, Laurent Billot, Lehana Thabane, Waleed Alhazzani, Adam Deane, Gordon Guyatt, Simon Finfer, François Lauzier, John Myburgh, Paul Young, Yaseen Arabi, John Marshall, Shane English, John Muscedere, Marlies Ostermann, Bala Venkatesh, Nicole Zytaruk, Miranda Hardie, Naomi Hammond, Serena Knowles, Lois Saunders, Alexis Poole, Abdulrahman Al-Fares, Feng Xie, Richard Hall, Deborah Cook

**Affiliations:** 1 Department of Health Research Methods Evidence and Impact, McMaster University, Hamilton, Ontario Canada; 2 The George Institute for Global Health, University of New South Wales, University of New South Wales Medicine & Health, Sydney, New South Wales Australia; 3 Department of Critical Care, Melbourne Medical School, University of Melbourne, Melbourne, Australia; 4 Critical Care Division, The George Institute for Global Health, University of New South Wales, Sydney, New South Wales Australia; 5 Division of Critical Care, Department of MedicineDepartment of Anesthesiology and Critical CareFaculty of Medicine, at l`Université LavalLaval UniversityUniversite Laval Faculte de medicine, Quebec, Canada; 6 Medical Research Institute of New Zealand, Wellington, New Zealand; 7 King Saud bin Abdulaziz University for Health Sciences, Riyad, Saudi Arabia; 8 Department of Surgery and Critical Care Medicine, Unity Health Toronto, University of Toronto, Toronto, Canada; 9 Department of Medicine (Critical Care), Faculty of Medicine, University of Ottawa, Ottawa, Canada; 10 Department of Critical Care Medicine, Queens University| Kingston Health Sciences Centre, Kingston, Ontario Canada; 11 Department of Critical Care, King’s College London, London, UK; 12 The George Institute for Global Health, University of New South Wales, Sydney, New South Wales Australia; 13 Academic Critical Care Office Room D176, Critical Care Medicine, St. Joseph’s Healthcare Hamilton, 50 Charlton Avenue East, Hamilton, Ontario Canada; 14 The George Institute for Global Health, Newton, Australia; 15 University of New South Wales, Sydney, New South Wales Australia; 16 The University of Adelaide, Adelaide, Australia; 17 Interdepartment Division of Critical Care Medicine, University of Toronto, Toronto, Canada; 18 Dalhousie University Faculty of Medicine, Halifax, Canada; 19 Department of Medicine, McMaster University, Hamilton, ON Canada

**Keywords:** Critically ill, Stress ulceration, Gastrointestinal bleeding, Ventilator-associated pneumonia, *Clostridioides difficile* infection

## Abstract

**Background:**

The REVISE (*R*e-*Ev*aluating the *I*nhibition of *S*tress *E*rosions in the ICU) trial will evaluate the impact of the proton pump inhibitor pantoprazole compared to placebo in invasively ventilated critically ill patients.

**Objective:**

To outline the statistical analysis plan for the REVISE trial.

**Methods:**

REVISE is a randomized clinical trial ongoing in intensive care units (ICUs) internationally. Patients ≥ 18 years old, receiving invasive mechanical ventilation, and expected to remain ventilated beyond the calendar day after randomization are allocated to either 40 mg pantoprazole intravenously or placebo while mechanically ventilated.

**Results:**

The primary efficacy outcome is clinically important upper GI bleeding; the primary safety outcome is 90-day mortality. Secondary outcomes are ventilator-associated pneumonia, *Clostridioides difficile* infection, new renal replacement therapy, ICU and hospital mortality, and patient-important GI bleeding. Tertiary outcomes are total red blood cells transfused, peak serum creatinine concentration, and duration of mechanical ventilation, ICU, and hospital length of stay. Following an interim analysis of results from 2400 patients (50% of 4800 target sample size), the data monitoring committee recommended continuing enrolment.

**Conclusions:**

This statistical analysis plan outlines the statistical analyses of all outcomes, sensitivity analyses, and subgroup analyses. REVISE will inform clinical practice and guidelines worldwide.

**Trial registration:**

www.ClinicalTrials.gov NCT03374800. November 21, 2017.

## Introduction

Approximately 2–4% of adults acutely admitted to an intensive care unit (ICU) develop upper gastrointestinal (GI) bleeding [1–4]. For bleeding prevention, approximately 70% of critically ill patients are prescribed stress ulcer prophylaxis [3], most commonly proton pump inhibitors (PPIs). In a recent European, multicenter, blinded trial, intravenous pantoprazole (PPI) was associated with a reduction in clinically important upper GI bleeding compared with placebo following an unplanned ICU admission of adults who were at risk for gastrointestinal bleeding [4]. Similarly, in PEPTIC, a large cluster-crossover trial, upper GI bleeding rates were lower during treatment periods when ICUs preferentially used PPIs for stress ulcer prophylaxis rather than histamine-2 receptor blockers (H_2_RAs) [5]. In both of these trials, upper GI bleeding was a secondary outcome; lack of adjustment for multiplicity, and high rates of non-adherence with study treatment respectively limited inferences from these observations. Additionally, among patients with high acuity of illness in both trials, there was a trend toward higher mortality in those receiving PPI. These findings highlight the need for further research to establish the efficacy and safety of PPIs for stress ulcer prophylaxis in contemporary ICU practice.

REVISE (Re-Evaluating the Inhibition of Stress Erosions) is an ongoing international randomized trial, the objective of which is to determine the effect of pantoprazole versus placebo on the primary *efficacy* outcome of clinically important upper GI bleeding and the primary *safety* outcome of 90-day mortality [[6]: NCT03374800]. Secondary outcomes are ventilator associated pneumonia (VAP), *Clostridioides difficile (C. difficile)* infection*,* renal replacement therapy, ICU and hospital mortality, and patient-important GI bleeding.

This document outlines the statistical analysis plan (SAP) for REVISE, reported using current guidelines [7].

## Methods

REVISE is a multicenter, randomized, stratified, concealed, blinded parallel group trial in patients ≥ 18 years old in the intensive care unit (ICU), designed in collaboration with the Canadian Critical Care Trials Group (CCCTG) [[8]:www.ccctg.ca], the Australian and New Zealand Intensive Care Society Clinical Trials Group (ANZICS-CTG) [[9]:www.anzics.com.au/clinical-trials-group], and other international colleagues. Inclusion and exclusion criteria are summarized in Table 1. In brief, eligible patients are adults who are admitted to the ICU, receiving invasive mechanical ventilation and expected to remain ventilated beyond the calendar day after randomization, and without a clear indication or contraindication to PPI. Eligible patients are enrolled by either a priori informed consent or by informed *consent to continue* or *deferred consent*, or *opt out*, in alignment with local approvals [10].

**Table 1:** Inclusion and exclusion criteria

**Inclusion criterion**
Adults ≥ 18 years old projected to receive invasive mechanical ventilation for ≥ 48 h according to the treating physician
**Exclusion criteria**
1. Already received invasive mechanical ventilation ≥ 72 h during this hospital admission 2. Acid suppression for active GI bleeding or high-risk of bleeding (e.g., current bleeding, peptic ulcer bleeding within 8 weeks, recent severe esophagitis, Barrett’s esophagus, Zollinger-Ellison syndrome) [dyspepsia or gastroesophageal reflux is not an exclusion criterion] 3. Acid suppression in the ICU for > 1 daily dose equivalent of a PPI or H2RA 4. Dual antiplatelet therapy 5. Combined antiplatelet and therapeutic anticoagulation 6. Pantoprazole contraindication per local product information 7. Palliative care or anticipated withdrawal of advanced life support 8, Pregnancy 9. Previous enrolment in REVISE, or a related trial, or trial for which coenrolment is prohibited 10. Patient, proxy, or physician declines

This table details the inclusion and exclusion criteria for the REVISE Trial. *ICU*, intensive care unit; *PPI*, proton pump inhibitor; *H2RA*, histamine-2-receptor antagonists

When notified by research staff or investigators about eligible patients, research pharmacists or other blinded study personnel use a password-protected website to access the automated central randomization program to ensure concealed 1:1 allocation, stratified by center and pre-hospital acid suppression (i.e., PPI or H_2_RA or neither). Randomized patients receive either 40 mg pantoprazole intravenously or an identical placebo while mechanically ventilated in the ICU up to 90 days after randomization or until death, discontinuation of mechanical ventilation, or clinically important GI bleeding. All patients, families, clinical, and research staff are blinded to allocation.

Research staff upload daily data from hospital charts to a secure web-based electronic data capture system (iDataFax, Seattle, Washington) including baseline data, study drug administration, daily data in ICU up to 90 days post randomization, relevant laboratory values, cointerventions, trial outcomes, reports for adjudication, duration of mechanical ventilation, ICU and hospital stay, and mortality. Patients discharged alive from hospital before 90 days are followed for 90 days to obtain vital status. Further details are reported elsewhere [[6]: NCT03374800] and in our protocol [10].

### Ethics

REVISE is approved by the Research Ethics Boards (REBs) and Human Research Ethics Committees (HRECs) of all participating regions and hospitals. REVISE is also approved by Health Canada [HC6-24-c210404], Clinical Trials Ontario Research Ethics Project ID: 1360, the Northern Sydney Local Health District Human Research Ethics Committee (HREC) [2019/ETH08405], and Comissão Nacional de Ética em Pesquisa (CONEP) [5.734.590]. Regulatory oversight in Canada is by Health Canada [HC6-24-c210404].

### Outcomes

*The primary efficacy outcome* is clinically important upper GI bleeding occurring in the ICU or resulting in ICU readmission during the index hospital stay [10]. Research staff prospectively collect data related to GI bleeding, allowing blinded central duplicate adjudication as described elsewhere [1, 2, 10].

*The primary safety outcome* is all-cause mortality at 90 days after randomization.

*Secondary outcomes* include VAP in the ICU, *C. difficile* infection in the hospital, new renal replacement therapy in the ICU, ICU and hospital mortality, and patient-important GI bleeding as defined through interviews and focus groups with ICU survivors and family members [11].

*Tertiary outcomes* include total red blood cells transfused in the ICU, peak serum creatinine concentration in the ICU, and duration of mechanical ventilation and duration of stay in the ICU and hospital, measured in days [10].

*Adverse events and serious adverse events* are as follows: key adverse events (AEs) and serious adverse events (SAEs) relevant to REVISE are already pre-defined primary or secondary trial outcomes. REVISE follows 5 published recommendations for rational reporting of SAEs in investigator-initiated ICU trials of drugs in common use [12].

### Sample size

The sample size of 4800 patients was chosen on the basis of plausible baseline risks of GI bleeding and plausible effect sizes of pantoprazole versus placebo and a target of 85% power [10]. Also, across the range of higher baseline risks of death due to high illness severity, 4800 patients will provide at least 70% power to detect an increase in risk of death associated with pantoprazole that would deter its use in such patients [4]. Further details are found elsewhere [[6]: NCT03374800] and in the REVISE protocol [10].

### Statistical analysis

All analyses will be conducted blinded to treatment allocation; the randomization code will only be broken when the analyses are complete, unless serious safety concerns arise. We will describe baseline characteristics of enrolled patients using descriptive statistics (count and percentage for categorical variables and mean or median with standard deviation or interquartile range for continuous variables, as appropriate).

Details regarding the main analysis plan, sensitivity analyses, and subgroup analyses are found in Table 2. The *main analyses* will be conducted per the intention-to-treat principle by analyzing patients according to their randomized group, regardless of protocol compliance. We will compare the time to the primary and secondary binary outcomes in the two groups using Cox proportional hazards regression. We will adjust for the stratification variable of pre-hospital acid suppression in the analyses [13, 14]. As the APACHE II score is strongly associated with mortality, to maximize statistical efficiency, we will also adjust for baseline APACHE II score for the mortality outcomes. For the primary efficacy and primary safety outcomes, we will present Kaplan-Meier curves. We will report hazard ratios with 95% confidence intervals (CIs) as well as the absolute risk differences and their respective 95% CIs. For the outcome of total red blood cells transfused in the ICU, we will compare the two groups using Poisson regression, adjusting for pre-hospital acid suppression. For all other continuous outcomes, we will compare the two groups using linear regression on the original scale or on the log-scale (to normalize the distribution, if needed), adjusting for pre-hospital acid suppression. If the distribution remains non-normal after transformation, we will employ a nonparametric approach. We will use graphics and other relevant methods to examine the assumption of proportional hazards for Cox regression analyses [15–17].

**Table 2:** Summary of analysis plan

**Objective**	**Outcome**	**Research hypothesis**	**Analysis method**^**†**^
**Main analysis of primary outcomes**			
To determine the effect of pantoprazole vs. placebo on the outcome	**Primary efficacy outcome:** Clinically important upper GI bleeding	Pantoprazole will reduce the risk of clinically important upper GI bleeding	Cox proportional hazards
	**Primary safety outcome:** All-cause 90-day mortality	Pantoprazole will increase the risk of death within 90 days	Cox proportional hazards, adjusted for APACHE II score
**Analysis of secondary outcomes**			
To determine the effect of pantoprazole vs. placebo on the outcome	VAP in ICU	Pantoprazole will increase the risk of VAP in ICU	Cox proportional hazards
	*C. difficile* infection in hospital	Pantoprazole will increase the risk of *C difficile* infection in hospital	Cox proportional hazards
	New treatment with renal replacement therapy in ICU	Pantoprazole will increase the risk of being treated with renal replacement therapy in ICU	Cox proportional hazards
	ICU mortality	Pantoprazole will increase the risk of death in ICU	Cox proportional hazards, adjusted for APACHE II score
	Hospital mortality	Pantoprazole will increase the risk of death in hospital	Cox proportional hazards, adjusted for APACHE II score
	Patient important upper GI bleeding	Pantoprazole will reduce the risk of patient important upper GI bleeding	Cox proportional hazards
**Analysis of tertiary outcomes**			
To determine the effect of pantoprazole vs. placebo on the outcome	Total units of red blood cells transfused in the ICU	Pantoprazole will have no effect	Poisson regression
	Peak serum creatinine concentration in the ICU	Pantoprazole will increase the peak serum creatinine concentration	Linear regression^‡^
	Duration of mechanical ventilation in days	Pantoprazole will have no effect	Linear regression^‡^
	Duration of ICU stay in days	Pantoprazole will have no effect	Linear regression^‡^
	Duration of hospital stay in days	Pantoprazole will have no effect	Linear regression^‡^
**Sensitivity analyses**			
To determine if the effect of pantoprazole vs. placebo is affected by not adjusting for pre-hospital acid suppression	**Primary efficacy outcome:** Clinically important upper GI bleeding	Analysis unadjusted for pre-hospital acid suppression will not change the effect of pantoprazole versus placebo on the risk of clinically important GI bleeding	Cox proportional hazards, not adjusted for pre-hospital acid suppression
	**Primary safety outcome:** All-cause 90-day mortality	Analysis unadjusted for pre-hospital acid suppression will not change the effect of pantoprazole versus placebo on the risk of death	Cox proportional hazards, adjusted for APACHE II score not adjusted for pre-hospital acid suppression
To determine if the effect of pantoprazole vs. placebo is affected by including center in the model as a random effect	**Primary efficacy outcome:** Clinically important upper GI bleeding	Analysis including center as a random effect will not change the effect of pantoprazole versus placebo on the risk of clinically important GI bleeding	Cox proportional hazards, including center as a random effect
	**Primary safety outcome:** All-cause 90-day mortality	Analysis including center as a random effect will not change the effect of pantoprazole versus placebo on the risk of death	Cox proportional hazards, adjusted for APACHE II score and including center as a random effect
To determine if the effect of pantoprazole vs. placebo is affected by the competing risk of death	**Primary efficacy outcome:** Clinically important upper GI bleeding	Accounting for the competing risk of death will not change the effect of pantoprazole versus placebo on clinically important GI bleeding	Fine and Gray proportional sub-distribution hazards model, with death as a competing risk
To determine the effect of pantoprazole vs. placebo under optimal protocol adherence as clinically appropriate (study drug received for ≥80% of days on mechanical ventilation)	**Primary efficacy outcome:** Clinically important upper GI bleeding	Pantoprazole will more greatly reduce the risk of clinically important GI bleeding than placebo in patients with maximal exposure	Cox proportional hazards
	**Primary safety outcome:** All-cause 90-day mortality	Pantoprazole will more greatly increase the risk of death than placebo in patients with maximal exposure	Cox proportional hazards, adjusted for APACHE II score
**Subgroup analyses**			
To determine if pre-hospital acid suppression modifies the effect of pantoprazole vs. placebo	**Primary efficacy outcome:** Clinically important upper GI bleeding	Pantoprazole is more effective in patients with pre-hospital acid suppression	Cox proportional hazards including an interaction term
	**Primary safety outcome:** All-cause 90-day mortality	No effect modification	Cox proportional hazards including an interaction term, adjusted for APACHE II score
To determine if APACHE II score modifies the effect of pantoprazole vs. placebo	**Primary efficacy outcome:** Clinically important upper GI bleeding	No effect modification	Cox proportional hazards including an interaction term
	**Primary safety outcome:** All-cause 90-day mortality	Pantoprazole is more harmful in patients with APACHE II score ≥25	Cox proportional hazards including an interaction term
To determine if medical admitting diagnosis modifies the effect of pantoprazole vs. placebo	**Primary efficacy outcome:** Clinically important upper GI bleeding	No effect modification	Cox proportional hazards including an interaction term
	**Primary safety outcome:** All-cause 90-day mortality	No effect modification	Cox proportional hazards including an interaction term, adjusted for APACHE II score
To determine if SARS-CoV-2 positive modifies the effect of pantoprazole vs. placebo	**Primary efficacy outcome:** Clinically important upper GI bleeding	No effect modification	Cox proportional hazards including an interaction term
	**Primary safety outcome:** All-cause 90-day mortality	Pantoprazole is more harmful in SARS-CoV-2 positive patients	Cox proportional hazards including an interaction term, adjusted for APACHE II score
To determine if sex modifies the effect of pantoprazole vs. placebo	**Primary efficacy outcome:** Clinically important upper GI bleeding	No effect modification	Cox proportional hazards including an interaction term
	**Primary safety outcome:** All-cause 90-day mortality	No effect modification	Cox proportional hazards including an interaction term, adjusted for APACHE II score

In this table, we outline planned analyses for the REVISE Trial, indicating the objective of each analysis, the outcome, research hypothesis, and analytic approach. For the Cox proportional hazards models, we will report hazard ratios and corresponding 95% confidence intervals. For the Poisson regression model, we will report incidence rate ratio and corresponding 95% confidence intervals. For the linear regression models, we will report mean differences and corresponding 95% confidence intervals. If the log-transformation or a nonparametric approach is used, we will report the *p*-value associated with the test, in addition to descriptive statistics (median, lower quartile, upper quartile) per group. *GI *gastrointestinal, *VAP *ventilator-associated pneumonia, *ICU *intensive care unit

^†^Randomization is stratified by pre-hospital acid suppression. All models adjust for this variable (except for the sensitivity analysis to determine if the effect of pantoprazole vs. placebo is affected by not adjusting for pre-hospital acid)

^‡^For continuous outcomes, we will compare the two groups using linear regression on the original scale or on the log-scale (to normalize the distribution, if needed). If assumptions are still not met after log-transformation, we will use nonparametric methods

Since all data are collected in hospital, except 90-day mortality for those discharged alive from hospital prior to 90 days, we anticipate very few missing data. For continuous outcomes with data missing for more than 2% of patients, we will perform multiple imputation analysis using multiple imputation by chained equations and will combine using Rubin’s rule [18]. Evidence has shown that multiple imputation is one of the optimal methods for handling data that is assumed missing at random [19]. For the time-to-event analyses, patients with incomplete follow-up will be censored at time of last follow-up.

All estimates of effect will be reported to two decimal places. *p*-values will be reported to three decimal places with those less than 0.001 reported as *p* < 0.001. The criterion for statistical significance will be set at alpha = 0.05, using 2-sided tests. Separately applied to secondary and tertiary outcomes, and to subgroup analyses, the sequential Holm-Šidák approach will be used to adjust for multiple significance testing [20, 21]. The sequential Holm-Šidák approach consists of ordering all *p*-values from smallest to largest, and then comparing them to an adjusted level of significance calculated as 1-(1-0.05)^1/*C*^, where *C* indicates the number of comparisons that remain. In the case of 11 outcomes, the smallest *p*-value would be compared to 1-(1-0.05)^1/11^, the second *p*-value to 1-(1-0.05)^1/10^, and so on, with the last one being compared to 1-(1-0.05) (i.e., 0.05). The sequential testing procedure stops as soon as a *p*-value fails to reach the corrected significance level [20, 21]. Analyses will be performed using SAS 9.4 (Cary, NC).

### Sensitivity analyses

We will perform sensitivity analyses as follows (Table 2).

- 1) For both the primary *efficacy* outcome (clinically important upper GI bleeding) and the primary *safety* outcome (90-day mortality), we will conduct a sensitivity analysis which is not adjusted for pre-hospital acid suppression.
- 2) For both the primary *efficacy* outcome (clinically important upper GI bleeding) and the primary *safety* outcome (90-day mortality), we will conduct a sensitivity analysis which includes center in the model as a random effect [22]. To avoid possible problems with model convergence due to a small number of events in some centers, we will not adjust for the stratification variable of center in the main models.
- 3) For our primary *efficacy* outcome (clinically important upper GI bleeding), we will conduct a sensitivity analysis using a competing risk analysis approach [23] with death as the competing risk using the Fine and Gray proportional sub-distribution hazards model [24, 25].
- 4) For both the primary *efficacy* outcome (clinically important upper GI bleeding) and the primary *safety* outcome (90-day mortality), we will conduct a sensitivity analysis restricted to patients receiving study drug on ≥ 80% of study days while mechanically ventilated, to investigate the effect of pantoprazole under conditions of optimal protocol adherence as clinically appropriate.

### Subgroup analyses

We will conduct analyses of both the primary *efficacy* outcome of clinically important upper GI bleeding and the primary *safety* outcome of 90-day mortality in five a priori subgroup pairs. Analyses will be adjusted for pre-hospital acid suppression. In Table 2, we pre-specify the hypothesized direction of effect, or lack thereof, also described here. For the overall trial, we hypothesize that pantoprazole will reduce the risk of clinically important upper GI bleeding and that pantoprazole will increase the risk of death within 90 days.

- 1) Pre-hospital use of acid suppression (PPIs or H2RAs) vs. none: We hypothesize that pantoprazole is more effective at preventing clinically important upper GI bleeding among patients with *versus* without pre-hospital acid suppression, given concerns about hypergastrinemia upon discontinuation of acid suppression [26, 27]. We hypothesize no modification of the effect of pantoprazole on 90-day mortality.
- 2) Illness severity measured by APACHE II score of ≥ 25 or < 25: We hypothesize no modification of the effect of pantoprazole on clinically important upper GI bleeding. We hypothesize that pantoprazole is more harmful in terms of increased risk of 90-day mortality among patients with higher versus lower illness severity, given the high illness severity subgroup finding in the SUPICU trial [4].
- 3) Medical vs. surgical/trauma ICU admitting diagnosis: We hypothesize no modification of the effect of pantoprazole on preventing clinically important upper GI bleeding. We hypothesize no modification of the effect of pantoprazole on 90-day mortality.
- 4) SARS-CoV-2 positive vs. negative status: We hypothesize no modification of the effect of pantoprazole on clinically important upper GI bleeding. We hypothesize that pantoprazole is more harmful in patients with versus without SARS-CoV-2 in terms of increased risk of 90-day mortality, given findings of prolonged course and worse outcomes of infected patients exposed to PPIs [28]. Patients with SARS-CoV-2 positive status will be considered those with active COVID-19 infection, not those with an incidental SARS-CoV-2 positive test result.
- 5) Female vs. male: We hypothesize no modification of the effect of pantoprazole on clinically important upper GI bleeding. We hypothesize no modification of the effect of pantoprazole on 90-day mortality. Sex is recorded for all patients, ascertained per medical chart documentation, and will also be analyzed and reported at baseline by randomized group. The sociocultural, environmental, and behavioral factors influencing a person’s self-identity according to gender are beyond the scope of this ICU trial.

We will include an interaction term between each subgroup variable and treatment group to test for subgroup effects. For any interaction *p*-value below 0.10, we will conduct a formal assessment of subgroup credibility using the only structured 4-category (high to very low credibility) instrument for assessing subgroup effects developed by an international expert panel, rigorously tested by multiple stakeholders with detailed guidance for each judgment [29].

### Interim analyses

The independent REVISE data monitoring committee (DMC) includes professor Roberts (Oxford), Dr. McAuley (Queen’s University of Belfast), and Dr. Thomlinson (Chair, University of Toronto). When 1200 patients were enrolled, the DMC reviewed 90-day mortality data and recommended continuing REVISE.

When 2400 patients (50% recruitment) had 90-day mortality ascertained, we conducted a single interim analysis of all outcomes. To maintain the overall type-I error rate (i.e., *α*), for the interim analysis, we used a conservative Haybittle-Peto stopping rule with a critical value of three standard deviations with a fixed conservative *α* = 0.001 [30, 31]. The DMC also examined reports regarding the following: (1) recruitment (center and patient), screening, consent, and coenrolment rates; (2) protocol procedures (randomization, stratification, study drug, and other adherence issues); and (3) trial outcomes.

Guided by their charter [32, 33], the DMC considered the balance of benefits and harms when reviewing the blinded interim analysis data. On December 5, 2022, the DMC advised the REVISE steering committee to continue enrolment to trial completion.

### Publication

The results manuscript will be submitted to a general clinical journal regardless of the results. Authorship will follow ICMJE guidelines. All trial sites, investigators, and research coordinators will be cited. Funders will have no influence on data handling, analysis, or writing of the manuscript. Our final report will follow guidance from CONSORT (Consolidated Standards of Reporting Trials) 2010 [34, 35] and CONSERVE (CONSORT and SPIRIT Extension for RCTs Revised in Extenuating Circumstances) [36]. The scientific protocol of REVISE was not modified due to the pandemic.

## Discussion

Ongoing uncertainty about the consequences of daily PPIs in critically ill patients have raised concerns about the risk-benefit ratio being shifted towards a neutral effect or harm. REVISE will provide low risk of bias estimates and more than double trial evidence on the impact of PPIs on health outcomes that will markedly increase the strength of inferences regarding clinically important bleeding, mortality, VAP, and *C. difficile* infection. Results will not necessarily be generalizable to patients who are breathing spontaneously or are receiving no enteral nutrition. Recruitment started on July 9, 2019. As of May 1, 2023, 4124 patients have been recruited in 63 centers internationally in Canada, Australia, the UK, USA, Saudi Arabia, Kuwait, and Pakistan.

Despite the low cost of pantoprazole per dose, acid suppression costs worldwide are sizable, given nearly universal daily prescribing in the ICU. Furthermore, integrating healthcare resources consumed for bleeding and infectious outcomes is necessary to inform policy. A parallel externally funded economic evaluation (E-REVISE) will address the cost-effectiveness of acid suppression in the ICU for healthcare systems. If acid suppression causes more harm than good or consumes resources without sufficient benefit, pantoprazole will be a candidate for de-adoption.

In this SAP, we have presented plans for using an intention-to-treat approach for analyses of all outcomes, including assessment of the robustness of the findings using a series of sensitivity analyses and subgroup analyses. We have followed the guidance for SAP for clinical trials to outline the analysis details of the trial [7]. Aligned with the World Health Organization's Joint Statement on Public Disclosure of Results from Clinical Trials, requiring timely public disclosure of results [37]:https://www.who.int/news/item/18-05-2017-joint-statement-on-registration, data will be analyzed and disseminated as soon as possible to inform bedside care and practice guidelines worldwide.

## Data Availability

Not applicable for this statistical analysis plan.
